# Factors influencing the professional quality of life in South African audiologists

**DOI:** 10.3389/fpsyg.2026.1735716

**Published:** 2026-08-06

**Authors:** Senamile G Ntuli, Ben Sebothoma

**Affiliations:** 1 Department of Speech-Language Pathology and Audiology, School of Health Care Sciences, Sefako Makgatho Health Sciences University, Pretoria, South Africa; 2 Department of Speech Pathology and Audiology, School of Human and Community Development, Faculty of Humanities, University of the Witwatersrand, Johannesburg, South Africa

**Keywords:** audiologists, professional quality of life, resilience, South Africa, workplace

## Abstract

**Background:**

Audiologists in South Africa operate within a complex, under-resourced healthcare system where high caseloads, limited institutional support, and emotional demands impact their professional quality of life. While international studies have explored the professional quality of life among various healthcare professionals, little is known about the contextual and psychosocial factors influencing the professional quality of life among audiologists, particularly in in low- and middle-income settings.

**Purpose:**

This study explored the factors influencing the professional quality of life of South African audiologists.

**Methods:**

A qualitative descriptive design was employed, involving semi-structured interviews with 15 South African audiologists who were purposively recruited. Data were analyzed thematically using Braun and Clarke, (2022) approach, guided by Resilience Theory, which conceptualizes resilience as a dynamic process of positive adaptation to adversity.

**Results:**

Six overarching themes emerged: (1) Compassion satisfaction and professional fulfillment from improving patients’ lives and mentoring others; (2) Burnout and emotional exhaustion: fatigue, frustration, and reduced empathy due to workload and resource constraints; (3) Secondary traumatic stress and compassion fatigue: emotional strain from repeated exposure to patient suffering and moral distress; (4) Resilience and coping mechanisms: adaptability, perseverance, teamwork, and emotional regulation that sustain professional functioning; (5) Support systems and organizational resources: the critical role of managerial guidance, teamwork, and mental health services; and (6) Personal and professional coping strategies: including rest, exercise, faith, therapy, and boundary-setting. These themes demonstrate how emotional labor, systemic challenges, and resilience interact to shape audiologists’ professional well-being.

**Conclusion:**

The study highlights the need for contextually responsive interventions that strengthen resilience, enhance peer and supervisory support, and address systemic barriers. Investing in organizational well-being and equitable resource allocation is essential to sustain the South African audiology workforce and improve care outcomes.

## Introduction

The professional quality of life (ProQOL) of healthcare professionals has emerged as a critical determinant of workforce well-being, job satisfaction, and patient outcomes (Moudatsou et al., 2020; Stamm, 2010). ProQOL represents the balance between the positive and negative psychological effects of helping others and is composed of three interrelated dimensions: compassion satisfaction (CS), the pleasure derived from doing one’s work well; burnout (BO), a state of emotional exhaustion and depersonalization; and secondary traumatic stress (STS), the stress that arises from indirect exposure to others’ trauma (Bride, 2007; Stamm, 2010). Together, burnout and secondary traumatic stress constitute compassion fatigue (CF), the cost of caring that can erode empathy, performance, and motivation. Maintaining a healthy ProQOL is therefore vital for sustaining practitioner well-being and ensuring quality of care (Ruiz-Fernández et al., 2021).

Within audiology, ProQOL is particularly relevant due to the emotional and communicative nature of the profession. Audiologists work closely with patients and families affected by hearing and communication disorders (Moeller et al., 2013; Zimmer et al., 2022), often managing diagnoses that have long-term social and emotional consequences (Moroe et al., 2025). The profession demands not only technical competence but also sustained empathy, patience, and emotional regulation. However, the emotional rewards of meaningful patient care are often tempered by systemic stressors, including heavy workloads, time pressures, and limited institutional support (Brito-Marcelino et al., 2020; Pillay et al., 2020).

Evidence from international studies have consistently shown that healthcare workers’ ProQOL is shaped by workplace conditions, organizational support, and individual coping strategies (Chung et al., 2020; Gonçalves et al., 2022; Kelly et al., 2015; Ruiz-Fernández et al., 2021; Zakeri et al., 2020). Healthcare professionals working in high-demand, resource-constrained environments often report lower compassion satisfaction and higher levels of burnout, while inadequate organizational resources and workplace support are associated with increased burnout and secondary traumatic stress (Chung et al., 2020; Gonçalves et al., 2022; Ruiz-Fernández et al., 2021). Compassion satisfaction has been linked to professional competence (Zakeri et al., 2020), and both professional maturity and supportive supervision appear to buffer emotional strain (De Andrade et al., 2024; Gonçalves et al., 2022).

Despite these insights, limited research exists on audiologists’ ProQOL, particularly in low- and middle-income countries (LMICs) (Blood et al., 2008; De Andrade et al., 2024; Severn et al., 2012; Zimmer et al., 2022). Studies in high-income settings suggest that early-career audiologists experience greater emotional exhaustion, depersonalization, and lower job satisfaction, often due to limited professional support and high emotional demands (Blood et al., 2008; Severn et al., 2012; Zimmer et al., 2022). However, the realities of audiological practice in LMICs are likely to differ due to additional contextual challenges, including workforce shortages, inadequate infrastructure, and socio-economic disparities (De Andrade et al., 2024; Moroe et al., 2025; Nagdee and Manuel de Andrade, 2023).

In South Africa, these challenges are compounded by a historically inequitable healthcare system, high patient volumes, and widespread resource constraints (Maphumulo and Bhengu, 2019). Audiologists, particularly those employed in the public sector, often face heavy caseloads, inadequate equipment, and limited access to mental health or supervisory support (Moroe et al., 2025; Sebothoma and Khoza-Shangase, 2021). Moreover, broader socio-economic stressors such as poverty, unemployment, and community violence, place an additional secondary pressure on healthcare providers serving these populations (Khoza-Shangase et al., 2022; Nagdee and Manuel de Andrade, 2023). These realities contribute to emotional fatigue and moral distress when clinicians are unable to meet patient needs due to structural limitations.

Resilience Theory (Van Breda, 2018) offers a valuable framework for understanding how audiologists navigate occupational challenges. Rather than viewing resilience as a static personality trait, the theory conceptualizes it as a dynamic process of positive adaptation to adversity, influenced by individual, relational, and environmental factors (Van Breda and Theron, 2018). Within the ProQOL framework, resilience is a key protective factor that enhances compassion satisfaction while reducing burnout and secondary traumatic stress (Gonçalves et al., 2022; Ruiz-Fernández et al., 2021). Resilient practitioners demonstrate emotional flexibility, self-efficacy, and meaning making, enabling them to sustain well-being despite systemic constraints (Lim and DeSteno, 2016; Gonçalves et al., 2022). However, in the context of South Africa’s public health system, where structural supports are lacking, individual resilience may be insufficient to counter persistent occupational stress. Consequently, there remains a significant gap in understanding the lived experiences of South African audiologists and the contextual factors that influence their professional quality of life, as existing studies have largely focused on quantitative assessments, offering limited insight into the nuanced psychological and organizational dynamics influencing well-being (De Andrade et al., 2024; Zimmer et al., 2022).

Given the limited qualitative evidence on professional quality of life among audiologists, particularly within LMICs (Blood et al., 2008; De Andrade et al., 2024; Khoza-Shangase et al., 2022; Nagdee and Manuel de Andrade, 2023; Zimmer et al., 2022), an interpretive qualitative approach was considered appropriate because it enables an in-depth exploration of lived experiences, perceptions, and the contextual factors shaping professional well-being. Such approaches are particularly valuable for investigating complex psychosocial phenomena that are embedded within organizational, interpersonal, and environmental contexts and cannot be adequately understood using quantitative measures alone (Braun and Clarke, 2022; Creswell and Poth, 2018). Although previous studies have documented burnout, compassion satisfaction, and resilience among healthcare professionals, little is known about how these experiences are shaped by systemic resource constraints and organizational factors within South African audiology practice.

Against this background, the present study aimed to explore the factors influencing the professional quality of life of South African audiologists, focusing on their emotional experiences, coping strategies, and perceptions of workplace support. By employing a qualitative approach grounded in resilience theory, this study seeks to generate an in-depth understanding of how audiologists interpret and navigate the emotional demands of their profession within a resource-constrained and socio-economically complex context. Such insights are essential for informing workforce development, policy reform, and the design of interventions that strengthen resilience and promote sustainable professional practice.

## Theoretical framework

This study is grounded in Resilience Theory, which provides a conceptual lens for understanding how individuals adapt and maintain well-being when confronted with adversity (Van Breda, 2018). Within this framework, resilience is viewed not as a fixed personal trait but as a dynamic and multidimensional process that enables positive adaptation through the interaction of individual, relational, and contextual factors. Resilience emerges through interactions between individual strengths, supportive relationships, and environmental resources that enable positive adaptation to adversity while sustaining psychological well-being and professional functioning (Van Breda and Theron, 2018).

In the context of ProQOL, resilience serves as a protective factor that mitigates the negative effects of occupational stress and enhances compassion satisfaction (Gonçalves et al., 2022; Ruiz-Fernández et al., 2021). High levels of resilience have been associated with reduced burnout and secondary traumatic stress, greater emotional regulation, and improved coping among healthcare professionals (Gonçalves et al., 2022; Ruiz-Fernández et al., 2021). Conversely, when resilience resources are depleted or unsupported by the organization, healthcare workers may experience compassion fatigue, moral distress, and professional disengagement (Coetzee and Laschinger, 2018).

Resilience Theory also acknowledges the contextual and systemic dimensions of adaptation. In resource-constrained healthcare systems such as South Africa’s, structural factors, including staffing shortages, inadequate supervision, and inequitable resource distribution, can limit professionals’ capacity to maintain resilience (Khoza-Shangase et al., 2022; Maphumulo and Bhengu, 2019). Consequently, resilience among South African audiologists cannot be understood solely at the individual level; it must also be examined within the organizational and socio-economic systems that either enable or constrain coping and recovery.

This study therefore adopts Resilience Theory to explore how audiologists construct meaning, manage emotional strain, and sustain professional engagement in the face of systemic and psychological challenges. By integrating this framework with the ProQOL model (Stamm, 2010), the study conceptualizes resilience as both a buffer against compassion fatigue and burnout, and a mechanism for enhancing compassion satisfaction and professional growth. This approach allows for a comprehensive understanding of how audiologists in South Africa navigate occupational stressors while striving to maintain both personal well-being and quality patient care.

## Materials and methods

### Research design

A qualitative descriptive design (Creswell and Creswell, 2018) was adopted to explore the factors influencing the ProQOL of South African audiologists. Semi-structured interviews were conducted to capture audiologists’ perspectives on factors that influence their professional quality of life, resilience, and coping mechanisms.

### Study setting

The study was conducted among practicing audiologists across South Africa, encompassing diverse work settings including public hospitals, community health centers, private practices, schools, and academic institutions. This national representation enabled the exploration of professional quality of life across a range of practice contexts and resource environments (Pillay et al., 2020). The inclusion of participants from both urban and rural settings further ensured that the data reflected the structural disparities inherent within the South African healthcare system (Khoza-Shangase et al., 2022; Maphumulo and Bhengu, 2019).

### Sampling and participants

A non-probability purposive sampling strategy was employed to recruit audiologists who could provide information-rich accounts of the factors influencing professional quality of life across diverse practice settings in South Africa. Purposive sampling was considered appropriate because it enabled the recruitment of participants with direct experience of the phenomenon while ensuring variation in practice setting, years of experience, and professional roles (Campbell et al., 2020).

Potential participants were recruited through professional associations (e.g., SAAA), social media platforms, and professional networks. Interested individuals contacted the researcher and were provided with a participant information sheet outlining the study purpose, participation requirements, and eligibility criteria. Participants were screened to confirm that they were registered with the Health Professions Council of South Africa (HPCSA) and were actively practicing as audiologists in South Africa at the time of the study. Eligible participants who provided written informed consent were subsequently invited to participate in an online interview scheduled at a mutually convenient time.

Eligible participants were audiologists registered with the HPCSA and actively practicing within South Africa at the time of the study, irrespective of nationality or ethnic background. Accordingly, the term “South African audiologists” in this study refers to audiologists practicing in South Africa rather than to nationality or ethnicity. Participants were employed across clinical, educational, academic, and research settings. Audiologists practicing outside South Africa and those qualified as audiologists but working exclusively as speech-language pathologists were excluded.

Recruitment continued until data saturation was achieved, as indicated by the absence of new themes emerging from the data (Braun and Clarke, 2022). The inclusion of participants from diverse practice settings was intentional to capture a broad range of experiences related to professional quality of life across the South African audiology workforce. Although work roles and organizational contexts differed across clinical, educational, academic, and research settings, these differences were treated as contextual influences rather than sources of bias. During analysis, themes were developed by identifying common patterns across participants while also considering setting-specific experiences where these contributed to participants’ perceptions of professional quality of life, resilience, and organizational support.

### Data collection instruments

Data were collected using a semi-structured interview guide (Adeoye-Olatunde and Olenik, 2021). The interview guide was developed based on the study objectives, the ProQOL framework, Resilience Theory, and the relevant literature. To establish content validity, the guide was reviewed by the research supervisors, who have expertise in audiology and qualitative research. Their feedback informed refinements to the wording, sequencing, and relevance of the interview questions to ensure alignment with the study objectives. The interview guide was subsequently pilot tested with two audiologists who met the inclusion criteria but were not included in the main study. Minor revisions were made to improve the clarity, flow, and comprehensibility of the questions before data collection commenced.

Open-ended questions were designed to explore audiologists’ experiences of professional fulfilment and stress, perceptions of workplace support, coping mechanisms, and the role of resilience in managing professional challenges. The interview schedule comprised five sections. The introductory section explained the purpose of the study, obtained informed consent, and outlined the interview process. The interview then explored participants’ (1) professional background and career motivations; (2) experiences of professional quality of life, including compassion satisfaction, burnout, and secondary traumatic stress; (3) workplace challenges, organizational support, and resilience; (4) the influence of professional and demographic factors on their experiences; and (5) coping strategies and recommendations for improving professional quality of life among South African audiologists. Open-ended questions encouraged participants to reflect on their experiences, while probing questions were used to clarify responses and explore emerging issues in greater depth. Representative questions included: “Can you describe aspects of your work that you find most rewarding or fulfilling?”, “Have you ever felt emotionally overwhelmed by a patient’s situation? How did you handle it?”, and “What personal strategies have you developed to manage the demands of your job?” The complete semi-structured interview guide is provided as Supplementary File 1.

Data were collected between October 2024 and December 2024 through individual semi-structured interviews conducted virtually via Microsoft Teams. Interviews lasted between 30 and 45 min. Online interviews were selected because they enabled the recruitment of audiologists from geographically diverse regions and practice settings across South Africa, thereby supporting the study aim of capturing a broad range of experiences. The virtual format also accommodated participants’ professional schedules while reducing geographical and logistical barriers to participation (De Villiers et al., 2021). Furthermore, online interviews have been shown to facilitate the collection of rich, in-depth qualitative data comparable to face-to-face interviews by promoting participant comfort, flexibility, and open discussion (Archibald et al., 2019).

All interviews were conducted in English because all participants were qualified audiologists with professional English proficiency. With participants’ written informed consent, interviews were audio-recorded and transcribed verbatim to ensure an accurate and comprehensive representation of participants’ accounts for thematic analysis.

### Data collection procedure

Ethical clearance was obtained from the University of the Witwatersrand Human Research Ethics Committee (Non-Medical) (Clearance No. H24/09/38). Participants were contacted individually via email to arrange interviews post giving consent. A pilot study was conducted with two audiologists from different work settings to test procedures. Their feedback informed minor adjustments to ensure clarity and relevance. A distress protocol (Draucker et al., 2009) was prepared to provide psychological support in case of emotional distress during interviews, though no participant required intervention.

### Researcher reflexivity

All interviews were conducted by the first author, an audiologist and academic with experience in clinical practice and qualitative research. The interviewer had no prior supervisory or professional relationship with the participants that may have influenced their decision to participate or their responses during the interviews. Recognizing that the researcher’s professional background and interest in workforce well-being could influence data interpretation, reflexivity was maintained throughout the study. Reflexive journalling was undertaken during data collection and analysis to document assumptions, methodological decisions, and emerging interpretations. Regular discussions with the supervisory team and peer debriefing were used to critically examine interpretations, challenge assumptions, and enhance the credibility and trustworthiness of the findings.

### Data analysis

Data were analysed using Braun and Clarke (2022) six-phase reflexive thematic analysis framework. Audio-recorded interviews were transcribed verbatim and checked for accuracy before analysis commenced. The researchers familiarized themselves with the data through repeated reading of the transcripts and documented initial impressions using reflexive memos.

Initial codes were generated inductively from participants’ accounts while remaining informed by the study aims and Resilience Theory. The first author conducted the initial coding of all interview transcripts using NVivo 14 to facilitate the systematic organization, management, and retrieval of the data. Conceptually related codes were grouped into preliminary themes, which were iteratively reviewed, refined, and discussed with the supervisory team. These discussions facilitated critical reflection on interpretations, refinement of conceptual boundaries, and the development of themes that accurately represented patterns across participants’ accounts. Consistent with Braun and Clarke (2022) Reflexive Thematic Analysis, themes were developed through an iterative and interpretive process rather than by seeking consensus or inter-coder agreement.

Themes were subsequently defined and named based on their ability to capture meaningful patterns across participants’ experiences and address the research objectives. Throughout the analysis, reflexive journalling and peer debriefing were used to critically examine how the researchers’ professional backgrounds, assumptions, and perspectives may have influenced data interpretation. Reflexive journalling and regular discussions with the supervisory team supported ongoing critical reflection and strengthened the credibility and confirmability of the findings.

### Trustworthiness

The trustworthiness of the study was established using the criteria of credibility, dependability, confirmability, and transferability (Nowell et al., 2017). Credibility was enhanced through the purposive recruitment of audiologists representing diverse professional backgrounds, years of experience, and practice settings. The interview guide was expert reviewed and pilot tested before data collection. During interviews, open-ended and probing questions elicited rich descriptions of participants’ experiences. Credibility was further strengthened through prolonged engagement with the data, peer debriefing, and the use of verbatim quotations to support the interpretation of findings.

Dependability was promoted through the systematic application of Braun and Clarke (2022) Reflexive Thematic Analysis. An audit trail documenting methodological decisions, coding development, theme refinement, and analytical decisions was maintained throughout the study to ensure transparency and methodological rigor. The use of NVivo 14 facilitated the systematic organization and management of the qualitative data.

Confirmability was strengthened through reflexive journalling and memo writing, enabling the researchers to critically examine their assumptions, perspectives, and potential influence on data collection and interpretation. Regular discussions among the research team facilitated critical reflection on emerging themes and helped ensure that the findings remained grounded in participants’ accounts.

Transferability was supported by providing detailed descriptions of the study context, participant characteristics, recruitment procedures, interview process, and analytical methods, allowing readers to determine the applicability of the findings to other healthcare and professional contexts. The inclusion of participants from diverse clinical, educational, academic, and research settings further enhances the potential transferability of the findings.

A completed Consolidated Criteria for Reporting Qualitative Research (COREQ) checklist (Tong et al., 2007) is provided as Supplementary File 2.

### Ethical considerations

Ethical principles outlined in the Declaration of Helsinki (World Medical Association, 2013) guided all procedures. Participants received a detailed participant information sheet about the study’s purpose, procedures, and potential risks and benefits before providing written informed consent. Participation was voluntary, and anonymity was maintained by assigning pseudonyms to transcripts. Data were securely stored on password-protected devices and accessible only to the research team. This study also adhered to the principles of autonomy, confidentiality, beneficence, and justice (Emanuel et al., 2004; Jelsma and Clow, 2005).

## Results

The demographic characteristics of the participants are presented in Table 1. The majority of participants were females (*n* = 14). Participants were predominantly early-career audiologists, with almost half reporting 0–5 years of clinical experience, and most holding an undergraduate bachelor’s degree (*n* = 10). The sample was heterogeneous in terms of work setting and ethnic background, reflecting the diversity of audiologists practicing in South Africa. Ethnicity was collected as a demographic characteristic to describe the diversity of the sample and was not used to define the study population or compare participant experiences. The predominance of early-career audiologists was considered during interpretation, as career stage may influence perceptions of professional quality of life, workplace stress, and coping experiences. The findings illustrate the complex interplay between personal resilience, systemic constraints, and workplace support shaping the professional well-being of South African audiologists.

**TABLE 1:** Participants’ demographic characteristics.

Participant’s ID	Highest qualification	Ethnicity	Age	Gender	Years of experience	Work setting
P6	Master’s	African	36–45	Female	11 to 15	Government Private
P8	Doctorate	African	46–55	Female	20+	Academic
P12	Bachelors	African	18–25	Female	0 to 5	Private
P21	Bachelors	Coloured	26–35	Female	0 to 5	Private
P25	Bachelors	White	18–25	Female	0 to 5	Private
P30	Bachelors	Indian	18–25	Female	0 to 5	Government
P33	Masters	Indian	26–35	Female	6 to 10	Academic Private
P34	Bachelors	African	36–45	Female	6 to 10	Private
P44	Bachelors	African	26–35	Female	6 to 10	Government
P49	Bachelors	African	18–25	Female	0 to 5	Government
P53	Bachelors	African	26–35	Female	0 to 5	Government
P76	Bachelors	White	36–45	Female	16 to 20	Government
P78	Doctorate	Prefer not to say	55+	Female	20+	Academic Private Government
P82	Bachelors	African	26–35	Female	0 to 5	Academic
P89	Doctorate	African	26–35	Male	11 to 15	Government

Analysis revealed two overarching categories comprising six themes and associated subthemes. Each theme and its associated subthemes are presented below and illustrated with representative participant quotations.

Factors Enhancing Professional Quality of Life

1. Compassion Satisfaction and Professional Fulfilment

2. Resilience and Coping Mechanisms

3. Support Systems and Organizational Resources

4. Personal and Professional Coping Strategies

Factors Diminishing Professional Quality of Life

5. Burnout and Emotional Exhaustion

6. Secondary Traumatic Stress and Compassion Fatigue

### Factors enhancing professional quality of life

#### Compassion satisfaction and professional fulfilment

This theme captures the positive aspects of professional quality of life, reflecting the sense of fulfilment, purpose, and motivation that participants derived from their work. Compassion satisfaction was associated with improving patients’ communication outcomes, enhancing quality of life, and, for participants in academic settings, contributing to the education of future audiologists.

#### Professional fulfilment

Participants expressed a strong sense of professional purpose and fulfilment derived from helping patients, witnessing progress, and contributing to the growth of the profession. Witnessing successful treatment outcomes reinforced their commitment to the profession and contributed to feelings of accomplishment. One participant explained, *That would be when I successfully complete a treatment. (P30, line 175).* Another reflected, *It just brings so much fulfilment because you can see this patient is benefiting from it. (P21, lines 136–137).* Similarly, another participant stated, *What makes it rewarding is the fact that you can see the difference that you’re making in someone’s life. (P25, lines 134–135).* These experiences illustrate that professional fulfilment was closely linked to participants’ ability to make a meaningful difference in the lives of their patients.

#### Positive patient outcomes and contributing to the profession

Beyond direct patient care, participants working in academic settings described satisfaction derived from educating and mentoring future audiologists. Supporting students was viewed as extending their impact beyond individual patients and contributing to the future of the profession. One participant explained, I’m training future audiologists who might do great things… If I’m training someone to be a good audiologist, it’s going to change many patients’ lives. (P33, lines 207–213). Another participant described the importance of supporting students during difficult periods, stating,

*I can be there for students when they need me… some students are dealing with so much. (P82, lines 112–114).*

Although compassion satisfaction was consistently evident, participants acknowledged that systemic challenges, including resource shortages and administrative demands, often limited their ability to experience these rewarding aspects of practice fully. Overall, compassion satisfaction emerged as an important protective aspect of professional quality of life, providing participants with a sense of purpose and reinforcing their commitment to the profession despite ongoing workplace challenges.

#### Resilience and coping mechanisms

This theme reflects the dynamic processes through which participants adapted to occupational challenges and maintained their professional well-being. Resilience was described as involving adaptability, perseverance, emotional regulation, and drawing on supportive interpersonal relationships to navigate workplace adversity.

#### Adaptability and perseverance

Participants frequently described resilience as the ability to continue functioning despite ongoing workplace demands. Rather than viewing resilience as an inherent personal characteristic, they described it as an active process of adapting to changing circumstances and finding practical solutions to challenges. One participant explained, Resilience is the ability to go on and it’s the ability to bounce back. (P25, lines 282–283). Others illustrated this adaptability by describing proactive responses to workplace challenges: The finances I’ve saved are depleting, so I started something that can generate income. (P12, line 220) I kept pushing until the company agreed to restart the hearing aid order. (P49, lines 233–234). These accounts illustrate participants’ capacity to adapt and persevere despite significant occupational pressures.

#### Emotional regulation

Participants also highlighted the importance of regulating their emotions to maintain professionalism during challenging clinical encounters. One participant explained, *You can’t get angry with the patient or… let them see that it’s affecting you.* (P30, lines 349–350). Another reflected, *It took resilience to accept that I can’t take this any further.* (P76, line 820). These findings suggest that resilience involved recognizing emotional limits while continuing to provide compassionate care.

#### Drawing on interpersonal support

Participants consistently described teamwork and supportive collegial relationships as important sources of resilience. Working within collaborative teams reduced feelings of isolation and enabled participants to cope more effectively with workplace challenges.

One participant stated, My team has been very supportive. (P44, line 152). Another observed, If we work together more, we would definitely improve our resilience (P76, line 820). Similarly, another participant described the benefits of a cohesive workplace culture: It’s a stable team where everyone knows everyone well. (P78, line 705). Collectively, these findings indicate that resilience was sustained through a combination of individual adaptability, emotional regulation, perseverance, and supportive interpersonal relationships. However, participants also recognized that resilience alone was insufficient to overcome persistent organizational and systemic challenges.

#### Support systems and organizational resources

This theme explores the influence of organizational and interpersonal support on participants’ professional quality of life. Participants consistently described organizational support as an important factor shaping their ability to cope with workplace demands, sustain resilience, and maintain professional well-being. Experiences varied depending on the availability of supportive leadership, collegial relationships, supervision, and employee well-being services.

#### Managerial support and leadership

Participants who experienced supportive managers described feeling reassured, valued, and better equipped to navigate challenging clinical situations. Responsive leadership created opportunities for open communication and fostered confidence in managing workplace demands.

One participant explained, *She helps me with what I’m supposed to do… that reassures me* (P21, lines 200–204). Another described how managerial support extended beyond administrative guidance to sharing emotionally demanding clinical responsibilities: *She started picking up when I just can’t with a patient anymore, and she’ll jump in* (P21, lines 439–440). Similarly, another participant highlighted the importance of approachable leadership, stating, *I always go to my line manager and say this is not working* (P33, lines 333–334). These experiences demonstrate that supportive leadership contributed to participants feeling psychologically safe, professionally supported, and more capable of managing workplace stress.

#### Collegial relationships and supervision

Participants also identified supportive colleagues and opportunities for supervision as valuable resources that reduced feelings of isolation and promoted professional well-being. Collaborative working environments encouraged shared problem-solving, emotional validation, and mutual support during challenging clinical situations.

Participants described supervision as extending beyond administrative oversight to include mentorship, guidance, and emotional support, particularly when navigating complex patient care or emotionally demanding situations.

#### Employee well-being services

While participants valued the availability of psychological support, they generally perceived these services as insufficient to meet the emotional demands of audiology practice. One participant explained, There’s only two therapists… you’ll only be seen within 4 months (P53, lines 323–330). Another participant described the available service, noting, It’s an institutional counselling and support kind of service… about eight sessions with a psychologist (P78, lines 586–588).

While the availability of psychological support was appreciated, participants felt that existing services were often insufficient to meet the emotional demands associated with audiology practice.

Overall, participants viewed organizational support as extending beyond material resources. Responsive leadership, effective supervision, supportive collegial relationships, and accessible well-being services were perceived as important protective factors that enhanced resilience and professional quality of life, whereas their absence contributed to workplace stress, emotional isolation, and diminished well-being.

#### Personal and professional coping strategies

This theme describes the practical strategies participants employed to manage occupational stress and maintain their emotional well-being. Participants adopted a range of personal and professional coping mechanisms, including self-care practices, establishing work-life boundaries, seeking psychological support, drawing on faith and spirituality, relying on supportive relationships, and advocating for organizational improvements.

#### Self-care practices

Participants frequently described engaging in activities that promoted physical and emotional recovery following demanding workdays. Sleep, rest, and exercise were commonly identified as important strategies for managing stress. One participant explained, Normally, I’d try to sleep. (P12, line 223). Another stated, I manage it with exercise. (P76, line 589). These practices helped participants restore emotional energy and maintain their overall well-being.

#### Work-life boundaries

Maintaining clear boundaries between work and personal life was considered essential for preventing occupational stress from extending into participants’ personal lives. One participant explained, I don’t take stuff home with me in my head. (P78, lines 336–337). Similarly another participant reflected, I’m very careful not to let work issues… consume me. (P6, lines 587–588). These findings suggest that consciously separating work from personal life was an important strategy for preserving emotional well-being.

#### Seeking psychological support and faith-based coping

Participants also described drawing on formal psychological support and personal belief systems to manage emotional challenges. One participant stated, *I’ve been in therapy for a while*. (P21, line 422). Another explained, *I’ve now been on an antidepressant… it’s just a game changer.* (P25, lines 329–330). Faith also emerged as an important source of emotional strength for some participants. As one participant shared, *I’m a Christian. So I pray*. (P12, lines 243–244). These findings illustrate that participants utilized both professional and personal resources to maintain psychological well-being.

#### Social support and workplace advocacy

Relationships with family, friends, and colleagues were consistently described as valuable sources of emotional support. One participant explained, Spending time with loved ones… helps me cope. (P30, lines 667–668). Another stated, I speak to my fellow colleagues and friends. (P34, lines 217–218). Similarly, one participant highlighted the reassurance provided through collegial support: They can reassure me that… the way you did this is good. (P21, lines 195–197). Participants also recognized the importance of organizational change in promoting professional well-being. Flexibility in workload and improved managerial communication were identified as strategies that could reduce occupational stress. One participant noted, I can choose whether I want to see a lot of patients… or just take it as an easy day. (P21, lines 234–236). Another recommended, Implement strategies to train managers… to make it easier for us to communicate. (P34, lines 337–338).

Collectively, these findings demonstrate that participants adopted a multifaceted approach to coping that combined personal self-care, emotional regulation, social support, and advocacy for organizational improvements. Although these strategies helped participants manage occupational stress, they were generally viewed as complementing rather than replacing the need for broader organizational and systemic support.

### Factors diminishing professional quality of life

#### Burnout and emotional exhaustion

This theme reflects participants’ experiences of prolonged occupational stress and its impact on their emotional well-being, motivation, and perceived professional effectiveness. Burnout was primarily attributed to chronic workplace demands, including high workloads, administrative responsibilities, staffing shortages, and resource limitations that hindered participants’ ability to deliver optimal care.

#### Emotional exhaustion

Participants consistently described feeling emotionally and physically depleted by the cumulative demands of their work. Persistent workloads, increasing administrative responsibilities, and staffing shortages contributed to feelings of fatigue, frustration, and diminished motivation. One participant reflected, Last year I was very burnt out and I was very, you know, frustrated, didn’t want to go to work anymore. The stress is high (P25, lines 68, 81–82). Another described how their enthusiasm for the profession had changed over time: When I just started off… I was excited for everything, but now toward the end it’s become a bit more like I just feel like I’m really tired all the time (P30, lines 211–213). Together, these accounts illustrate the cumulative psychological burden imposed by sustained organizational demands.

#### Depersonalization

As workplace pressures intensified, some participants described becoming emotionally detached from their work and from the patients they served. Participants viewed this emotional distancing as an unintended consequence of prolonged stress rather than a deliberate reduction in compassion. One participant explained, We have quite a high outpatient load… I tend to get detached, I guess I do also feel that. (P30, lines 215–216, 248–249). Participants suggested that this emotional detachment enabled them to continue functioning in demanding clinical environments, although they recognized its potential impact on professional engagement.

#### Reduced professional efficacy

Participants also expressed frustration when resource shortages, inadequate equipment, and staffing constraints prevented them from providing the standard of care they believed patients deserved. Rather than describing the emotional impact of patient suffering, participants emphasized the professional dissatisfaction associated with being unable to perform their roles effectively. One participant stated, With government… the shortage of hearing aids was challenging. It was prohibiting me from performing my best (P33, lines 152–153, 247, 513–514). Similarly, another participant stated, The lack of resources prevents me from being the best at my job professionally (P89, lines 324–325). Collectively, these findings indicate that burnout was primarily driven by chronic organizational pressures that depleted participants’ emotional energy, reduced motivation, and limited their capacity to practice effectively.

#### Secondary traumatic stress and compassion fatigue

This theme describes the emotional consequences of repeated exposure to patients’ distress and the psychological burden associated with caring for individuals experiencing significant communication and social challenges. Unlike burnout, which was linked primarily to chronic workplace conditions, secondary traumatic stress and compassion fatigue arose from participants’ emotional engagement with patients and the moral distress associated with providing care under constrained circumstances.

#### Emotional investment in patients

Participants described becoming emotionally affected by the difficult circumstances faced by their patients, particularly children and families experiencing significant adversity. Many described difficulty maintaining emotional distance from patients’ experiences. One participant explained, The most emotionally challenging because you become so invested… because they’re children (P76, lines 495–496). Another reflected, Even if you try not to, you just feel for them (P30, line 291), while another stated, I’ve heard some pretty horrendous things… that have been difficult for me emotionally (P78, lines 304–305). These accounts demonstrate the emotional investment that characterized participants’ relationships with their patients.

#### Moral distress

Participants also described experiencing moral distress when systemic barriers prevented them from providing timely or appropriate care. Rather than the resource shortages themselves being the primary source of distress, participants emphasized the emotional burden of knowing what patients required but being unable to provide it. One participant explained, *Sometimes you don’t have the equipment or the supplies that you need in order to help everyone* (P53, lines 62–63). Similarly, another reflected, *It becomes a little not so satisfying ‘cause it’s a case of… since this is a public hospital, we have to place you on the waiting list* (P30, lines 132–134). These findings illustrate how resource limitations contributed to emotional distress by preventing participants from delivering the care they believed their patients deserved.

#### Compassion fatigue

Participants recognized that sustained empathic engagement with distressed patients could gradually diminish their emotional capacity. Compassion fatigue was described as the cumulative emotional toll of repeatedly supporting patients experiencing complex communication, health, and social challenges. One participant noted, *It does cause a bit more stress because sometimes if the patient is a bit difficult… you have to deal with them, be patient as the professional* (P30, lines 347–349). Another observed, *They tend to get empathy fatigue and then just stagnate* (P76, lines 796–797). A further participant linked these emotional demands to the realities of working within constrained healthcare environments: *It’s overwhelming because we have long waiting lists and we are short-staffed* (P34, lines 197–198).

Overall, these findings demonstrate that secondary traumatic stress and compassion fatigue were driven primarily by participants’ emotional engagement with patients and the moral burden associated with providing compassionate care within resource-constrained environments. While burnout reflected the cumulative impact of chronic organizational stressors, secondary traumatic stress and compassion fatigue arose from the emotional demands inherent in caring for patients experiencing significant communication and psychosocial challenges.

## Discussion

This study explored the factors influencing the professional quality of life of South African audiologists through in-depth qualitative interviews. Guided by Resilience Theory (Van Breda, 2018), the findings demonstrate that professional quality of life is shaped by dynamic interactions among individual, interpersonal, organizational, and systemic influences. Participants described both protective experiences that enhanced professional well-being and workplace challenges that diminished it, illustrating the complex and multidimensional nature of professional quality of life within the South African healthcare context.

### Compassion satisfaction and professional fulfilment

The theme of Compassion Satisfaction and Professional Fulfilment highlights the positive dimensions of audiologists’ professional quality of life. The subthemes of professional fulfilment, positive patient outcomes, and contributing to the profession demonstrate that participants derived meaning from improving patients’ communication and quality of life, witnessing successful clinical outcomes, and supporting the education and development of future audiologists. These experiences strengthened participants’ professional identity, reinforced their sense of purpose, and functioned as important protective factors against occupational stress.

These findings align with Stamm (2010) conceptualization of compassion satisfaction as the pleasure derived from helping others and achieving positive professional outcomes. Participants consistently described moments of fulfilment when patients regained hearing, communication, or participation in everyday life, reaffirming their commitment to audiology despite the emotional demands of practice. Similarly, participants working in academic settings experienced satisfaction from mentoring students and contributing to the future of the profession, suggesting that compassion satisfaction extends beyond direct patient care to encompass broader professional contributions.

However, compassion satisfaction was not experienced in isolation. Participants frequently described how systemic inefficiencies, long waiting lists, workforce shortages, and limited access to clinical resources constrained their ability to provide timely and effective care. Consequently, professional fulfilment was often tempered by frustration when organizational barriers prevented participants from delivering the standard of care they believed patients deserved. These findings suggest that while compassion satisfaction serves as an important protective resource, its sustainability depends not only on individual motivation but also on organizational and health system conditions that enable clinicians to practice effectively. Similar findings have been reported among South African healthcare professionals, who continue to derive meaning from their work despite practicing within under-resourced environments (Khoza-Shangase et al., 2022; Moroe et al., 2025).

### Resilience and coping mechanisms

The theme of Resilience and Coping Mechanisms illustrates the dynamic processes through which audiologists adapted to occupational challenges while maintaining their professional well-being. The subthemes of adaptability, perseverance, emotional regulation, and drawing on interpersonal support demonstrate that resilience was not perceived as a fixed personality trait but as an evolving process influenced by interactions between individual capacities and environmental resources.

Participants described adaptability as essential for responding to changing workplace demands and navigating the realities of practicing within resource-constrained environments. Flexibility, acceptance, and problem-solving enabled participants to continue providing patient care despite persistent organizational challenges. Perseverance was similarly linked to participants’ commitment to their profession and their desire to continue delivering meaningful care despite adversity. These findings suggest that resilience was closely intertwined with compassion satisfaction, reinforcing participants’ motivation to remain engaged in their work.

Participants also emphasized the importance of emotional regulation, recognizing the need to manage emotional responses, maintain professional boundaries, and prevent workplace stress from negatively affecting patient care or personal well-being. Importantly, resilience was consistently described as being strengthened through supportive relationships with colleagues, family members, mentors, and faith communities. Rather than relying solely on individual determination, participants viewed resilience as a shared process sustained through interpersonal connections.

These findings are consistent with previous research identifying resilience as a protective factor associated with greater compassion satisfaction and reduced burnout among healthcare professionals (Coetzee and Laschinger, 2018; Gonçalves et al., 2022; Lim and DeSteno, 2016). They also support Van Breda (2018) conceptualization of resilience as a dynamic interaction between individuals and their environments rather than an inherent personal characteristic. Importantly, participants acknowledged the limits of resilience when organizational support was lacking, suggesting that resilience should not be interpreted as an expectation that clinicians simply endure increasingly difficult working conditions. Instead, resilience requires workplace environments that actively promote psychological well-being, supportive leadership, and equitable access to resources.

### Support systems and organizational resources

The theme of Support Systems and Organizational Resources underscores the important role of organizational and interpersonal support in shaping audiologists’ professional quality of life. The subthemes of managerial support, collegial relationships, supervision, and employee well-being services demonstrate that supportive workplace environments buffer the effects of occupational stress and strengthen resilience.

Participants consistently described approachable managers, collaborative colleagues, and supportive supervisors as important sources of reassurance during challenging clinical situations. These organizational relationships promoted psychological safety, facilitated problem-solving, and encouraged open communication, enabling participants to manage workplace demands more effectively. For many participants, supportive colleagues also provided opportunities for informal debriefing and emotional validation following difficult clinical encounters.

Conversely, participants highlighted important gaps in organizational support. Although some institutions offered counselling services and employee assistance programmes, these services were often perceived as difficult to access because of limited availability and lengthy waiting periods. As a result, participants frequently relied on informal peer support rather than formal organizational well-being initiatives.

These findings are consistent with previous research demonstrating that perceived organizational support enhances compassion satisfaction while reducing burnout and emotional exhaustion (Chung et al., 2020; Ruiz-Fernández et al., 2021). Within the context of Resilience Theory, organizational support functions as an external protective resource that strengthens individuals’ capacity to adapt to workplace adversity (Van Breda, 2018). The findings therefore suggest that strengthening leadership, supervision, and employee well-being programmes may be as important as developing individual resilience in promoting sustainable professional quality of life.

### Personal and professional coping strategies

Participants described a range of personal and professional coping strategies that enabled them to manage occupational stress and maintain their emotional well-being. Consistent with the findings, these strategies included establishing work-life boundaries, engaging in self-care activities, seeking psychological support, drawing on faith and spirituality, and relying on supportive relationships with colleagues, friends, and family. Rather than representing isolated behaviors, these coping strategies complemented the resilience processes described above by enabling participants to regulate emotional demands and sustain professional engagement despite ongoing workplace challenges.

These findings are consistent with previous research demonstrating that adaptive coping strategies are associated with greater resilience, higher compassion satisfaction, and lower levels of burnout among healthcare professionals (Gonçalves et al., 2022; Ruiz-Fernández et al., 2021). However, participants also recognized that individual coping strategies could only partially offset persistent organizational and systemic challenges. This finding reinforces the importance of complementing individual resilience-building initiatives with organizational interventions that address the root causes of occupational stress rather than relying solely on individuals to adapt to increasingly demanding work environments.

### Burnout and emotional exhaustion

The theme of Burnout and Emotional Exhaustion captures the cumulative impact of chronic workplace demands on participants’ emotional well-being, motivation, and perceived professional effectiveness. The subthemes of emotional exhaustion, depersonalization, and reduced professional efficacy illustrate how persistent organizational pressures, including excessive workloads, staffing shortages, administrative responsibilities, and resource limitations, contributed to participants’ experiences of burnout.

Participants described feeling emotionally and physically exhausted after prolonged exposure to demanding work environments. Many reflected on a gradual decline in motivation and enthusiasm for their work as occupational pressures accumulated over time. These findings are consistent with the conceptualization of burnout as a syndrome characterized by emotional exhaustion, depersonalization, and diminished professional accomplishment resulting from chronic occupational stress (Maslach and Leiter, 2016).

Importantly, participants’ experiences of burnout were largely attributed to organizational rather than patient-related factors. High patient volumes, insufficient staffing, increasing administrative responsibilities, and inadequate clinical resources created workplace environments that made it difficult to provide the quality of care participants aspired to deliver. Resource shortages not only increased workloads but also undermined participants’ sense of professional competence when they were unable to perform their roles effectively. These findings are consistent with the Job Demands–Resources (JD-R) model, which proposes that excessive job demands in the absence of adequate organizational resources increase the risk of burnout and psychological strain (Bakker and Demerouti, 2007).

The findings also reflect broader workforce challenges within the South African healthcare system, where shortages of personnel, equipment, and financial resources continue to place considerable pressure on healthcare professionals (Pillay et al., 2020; Khoza-Shangase et al., 2022). Similar experiences have been reported among audiologists internationally, with workload pressures, administrative burden, and insufficient organizational support consistently associated with higher levels of burnout and reduced professional quality of life (Zimmer et al., 2022; Severn et al., 2012). Collectively, these findings suggest that reducing burnout requires organizational interventions that address workload, staffing, and resource allocation alongside initiatives that promote employee well-being.

### Secondary traumatic stress and compassion fatigue

The theme of Secondary Traumatic Stress and Compassion Fatigue reflects the emotional consequences of sustained empathic engagement with patients experiencing hearing loss, communication difficulties, and associated psychosocial challenges. Unlike burnout, which was primarily associated with chronic organizational pressures, the subthemes of emotional investment, moral distress, and compassion fatigue demonstrate that secondary traumatic stress arose from participants’ close emotional engagement with patients and the psychological burden of providing care within resource-constrained environments.

Participants described becoming emotionally invested in their patients’ experiences, particularly when working with children and families facing significant adversity. Many reported that repeated exposure to patients’ distress resulted in emotional strain and psychological fatigue, despite efforts to maintain professional boundaries. These findings are consistent with Stamm (2010) conceptualization of secondary traumatic stress as the emotional impact of indirect exposure to the trauma and suffering of others through professional caregiving.

Participants also described experiencing moral distress when systemic constraints prevented them from providing timely or appropriate care. Rather than the lack of resources itself being the primary source of distress, participants emphasized the emotional burden of knowing what patients required while being unable to provide it because of equipment shortages, long waiting lists, or service limitations. These findings highlight an important distinction between burnout and secondary traumatic stress. Whereas burnout reflected the cumulative effects of organizational pressures, secondary traumatic stress was characterized by the emotional burden associated with patients’ suffering and the frustration of being unable to meet their needs.

Compassion fatigue emerged as the cumulative consequence of sustained empathic engagement with patients over time. Participants recognized that continually supporting individuals experiencing communication difficulties and psychosocial adversity could gradually diminish their emotional capacity, leading to empathy fatigue and emotional depletion. Previous studies similarly report that repeated exposure to patients’ distress increases the risk of secondary traumatic stress and compassion fatigue among healthcare professionals (Kumar et al., 2024; Stamm, 2010; Ruiz-Fernández et al., 2021). These findings reinforce the need for structured opportunities for debriefing, reflective practice, and psychological support to minimize the emotional impact of caring for vulnerable patient populations.

An important contextual consideration when interpreting these findings is that a substantial proportion of participants were early-career audiologists with 0–5 years of clinical experience. Although the study was not designed to compare experiences across career stages, participants’ professional experience may have influenced their perceptions of professional quality of life. Early-career practitioners often navigate the transition from academic training to independent clinical practice while simultaneously developing professional confidence, managing increasing workplace responsibilities, and establishing effective coping strategies.

This interpretation is supported by previous research, which suggests that early-career audiologists are more likely to experience emotional exhaustion, workplace stress, and reduced job satisfaction while developing professional competence and support networks (Blood et al., 2008; Severn et al., 2012; Zimmer et al., 2022). Within the present study, some experiences of burnout, uncertainty, and resilience may therefore reflect not only the structural challenges associated with practicing in the South African healthcare system but also the developmental realities of the early stages of professional practice. Future research comparing audiologists across different career stages may provide further insight into how professional quality of life evolves throughout the professional lifespan.

Another important contextual consideration is that participants represented diverse practice settings, including public healthcare, private practice, educational institutions, and academia. Despite this diversity, several experiences were remarkably consistent across contexts. Regardless of practice setting, participants described common challenges related to emotional labor, professional fulfilment, resilience, and the need for organizational support, suggesting that many determinants of professional quality of life transcend specific employment sectors.

At the same time, some contextual differences were evident. Participants working in public-sector settings more frequently highlighted resource shortages, staffing constraints, and high patient volumes, whereas those in academic settings emphasized student supervision, mentoring responsibilities, and balancing teaching, research, and, in some instances, clinical commitments. These findings indicate that while the core dimensions of professional quality of life were shared across settings, the organizational and contextual factors shaping these experiences varied according to the nature of the work environment. Rather than representing a source of bias, the inclusion of participants from diverse practice settings strengthened the study by capturing the breadth and complexity of experiences that characterize the audiology profession in South Africa. This diversity enhanced the transferability of the findings by demonstrating how common aspects of professional quality of life manifest across different professional contexts.

Collectively, these findings extend the application of Resilience Theory (Van Breda, 2018) to the South African audiology workforce by demonstrating that professional quality of life is shaped through the dynamic interaction of individual, interpersonal, organizational, and systemic factors. Compassion satisfaction emerged as an important protective resource that fostered professional meaning and purpose, while burnout and secondary traumatic stress reflected the cumulative effects of chronic workplace demands and sustained emotional engagement with patients. Organizational support, collegial relationships, supervision, accessible employee well-being services, and adaptive coping strategies functioned as protective resources that strengthened participants’ capacity to adapt, whereas persistent organizational and systemic constraints undermined their professional well-being. Importantly, these findings demonstrate that resilience should not be viewed as an individual responsibility or an expectation that clinicians simply endure increasingly difficult working conditions. Rather, resilience develops through the interaction between individuals and their environments and therefore depends upon organizational structures that promote psychological well-being, supportive leadership, equitable access to resources, and sustainable working conditions.

Beyond confirming the established dimensions of the ProQOL framework, this study offers new insight into how these dimensions are experienced and negotiated within a resource-constrained healthcare context. Rather than identifying burnout, secondary traumatic stress, and compassion satisfaction as isolated outcomes, the findings demonstrate that these experiences are dynamically shaped by systemic resource shortages, organizational support, and the moral demands of clinical practice. Participants frequently described distress arising not only from heavy workloads but from knowing what patients required while being unable to provide optimal care because of equipment shortages, long waiting lists, staffing constraints, and broader health system limitations. These findings suggest that moral distress may represent an important mechanism through which structural healthcare challenges influence professional quality of life among audiologists in low- and middle-income settings. Furthermore, the study demonstrates that resilience is not simply an individual attribute but is co-constructed through supportive leadership, collegial relationships, supervision, and organizational resources. By highlighting these interconnected organizational and systemic influences, this study extends the application of the ProQOL framework beyond individual psychological experiences and contributes contextually relevant evidence for understanding workforce well-being within resource-constrained healthcare systems.

The findings have several important implications for policy, practice, and professional training. Healthcare organizations should prioritize accessible psychological support services, structured clinical supervision, mentorship programmes, and supportive leadership practices to strengthen workforce well-being. Attention should be directed toward early-career audiologists, who may benefit from additional mentorship and structured transition programmes as they develop professional competence and resilience. Educational programmes should continue to integrate resilience-building, reflective practice, self-care, and professional well-being into undergraduate and postgraduate curricula to better prepare graduates for the emotional demands of clinical practice. At a broader systems level, addressing workforce shortages, improving access to clinical resources, and strengthening organizational support structures are likely to enhance both professional quality of life and workforce sustainability within the South African audiology profession.

### Limitations

Several limitations should be acknowledged. This qualitative study included a relatively small, purposively selected sample of audiologists. Although participants represented diverse professional contexts, the findings are intended to provide an in-depth understanding of participants’ lived experiences within the South African context rather than to be statistically generalizable. The sample was predominantly female and included a substantial proportion of early-career audiologists, which may have influenced the perspectives captured and may limit the transferability of the findings to male audiologists and more experienced practitioners. In addition, the use of self-reported data may have been influenced by social desirability bias or participants’ willingness to disclose emotionally challenging experiences. Finally, although all participants were professionally proficient in English, conducting the interviews exclusively in English may have limited the expression of nuanced emotional experiences for participants whose first language was not English. Readers should therefore consider the contextual nature of these findings when assessing their applicability to other professional and healthcare settings.

### Recommendations for future research

Future research should employ longitudinal and intervention-based designs to examine how professional quality of life evolves over time and in response to targeted support initiatives. Comparative studies across public, private, academic, and educational settings would further clarify how organizational environments, resource availability, and work demands shape professional quality of life. Cross-professional research involving other rehabilitation and healthcare disciplines could identify shared and profession-specific resilience processes and inform interdisciplinary workforce support strategies. Finally, the development and validation of culturally responsive measures of compassion satisfaction, burnout, secondary traumatic stress, and resilience would strengthen the applicability of ProQOL frameworks within African healthcare contexts.

## Conclusion

This study provides an in-depth understanding of the factors shaping the professional quality of life of South African audiologists within diverse practice settings. Participants described a dynamic interplay between compassion satisfaction, burnout, secondary traumatic stress, resilience, organizational support, and systemic healthcare challenges. Although many audiologists demonstrated resilience and a strong sense of professional purpose, persistent organizational and structural constraints continued to influence their well-being.

These findings reinforce the importance of viewing professional quality of life as the product of interactions between individual, organizational, and broader health system factors rather than as an individual responsibility alone. Strengthening organizational support, promoting supportive leadership, improving access to psychological well-being services, and addressing workforce and resource constraints are likely to enhance both workforce well-being and the long-term sustainability of the South African audiology profession.

## Data Availability

The raw data supporting the conclusions of this article will be made available by the authors, without undue reservation.
