# Supplementary material for: Factors influencing the professional quality of life in South African audiologists
Source: Front Psychol. 2026 Aug 6;17:1735716. doi: 10.3389/fpsyg.2026.1735716 (PMC13489920; doi:10.3389/fpsyg.2026.1735716)
Supplement: Supplementary file 1 [file Data_Sheet_1.pdf]

Interview Guide

Introduction

- **Welcome and Overview**:
    - Welcome and thank the participant for their time.
    - Explain the purpose of the interview and the study:
        - "We're exploring the professional quality of life of South African audiologists, focusing on experiences such as compassion satisfaction, compassion fatigue, burnout, and resilience."
    - Obtain informed consent:
        - Explain confidentiality, data storage, anonymity, and participants' rights, including the ability to withdraw at any time.
        - Confirm understanding and address any questions before starting.
    - Thank participants for providing audio recording consent.
- **Outline the Interview Process**:
    - "The interview will begin with a few background questions, followed by more in-depth discussions about your experiences, challenges, and strategies related to your professional life."

Opening Questions

1. "Can you tell me a bit about your professional background and current role?"
2. "What inspired you to pursue a career in audiology?"

Main Questions

Topic 1: Professional Quality of Life

1. "How would you describe your overall experience working in your current role?"
2. "Can you describe aspects of your work that you find most rewarding or fulfilling (compassion satisfaction)?"
3. "Do you ever feel emotionally exhausted or detached at work? Can you share an example (burnout)?"
4. "Have you ever felt emotionally overwhelmed by a patient's situation? How did you handle it (secondary traumatic stress)?"

Topic 2: Stress and Challenges

5. "Can you describe a particularly challenging situation you've faced in your work?"
6. "How do these challenges affect your overall feelings of satisfaction or stress in your role?"
7. "What resources or support systems are available to help you manage these challenges?"

Topic 3: Resilience

8. "What does resilience mean to you in your professional life?"
9. "Can you describe a time when your resilience helped you cope with a challenging situation at work?"
10. "How do you think resilience influences your ability to balance the rewarding and stressful aspects of your job?"

Topic 4: Association with Demographics

12. "How do you think factors like your years of experience or work setting (e.g., rural vs. urban) influence your professional quality of life?"
13. "Do you feel that personal factors like age, gender, or family responsibilities play a role in how you experience stress or satisfaction at work?"

Topic 5: Resources and Strategies

14. "What resources or support systems are currently available to improve your professional quality of life?"
15. "What additional resources or changes do you think could better support audiologists in South Africa?"
16. "What personal strategies have you developed to manage the demands of your job?"
17. "If you could recommend one change to improve the quality of life for audiologists in South Africa, what would it be?"

Probing Questions

- "Can you elaborate on that experience?"
- "What impact did that situation have on your professional life?"

- "How do you think this compares to the experiences of other audiologists?"

Closing Questions

1. "Is there anything else you would like to add about your professional quality of life?"
2. "Do you have any suggestions for improving resilience or professional satisfaction among audiologists in South Africa?"

Conclusion

- Thank the participant for their time and valuable insights.
- Provide an overview of the next steps:
  - "We'll analyze the data over the next few months, and results will be shared in a summarized format. Let us know if you'd like a copy of the final findings."
- Provide your contact information for any follow-up questions or concerns.
