# Supplementary material for: Factors influencing the professional quality of life in South African audiologists
Source: Front Psychol. 2026 Aug 6;17:1735716. doi: 10.3389/fpsyg.2026.1735716 (PMC13489920; doi:10.3389/fpsyg.2026.1735716)
Supplement: Supplementary file 2 [file Data_Sheet_2.pdf]

Consolidated criteria for reporting qualitative studies (COREQ): 32-item checklist

Developed from:
Tong A, Sainsbury P, Craig J. Consolidated criteria for reporting qualitative research (COREQ): a 32-item checklist for interviews and focus groups. *International Journal for Quality in Health Care*. 2007. Volume 19, Number 6: pp. 349 – 357

MANUSCRIPT TITLE: Factors influencing the professional quality of life in South African audiologists

No. Item	Guide questions/description	Reported on Page #
**Domain 1: Research team and reflexivity**		
Personal Characteristics		
1. Inter viewer/facilitator	Which author/s conducted the interview or focus group?	First author (Senamile Ntuli)
2. Credentials	What were the researcher's credentials? (E.g. PhD, MD)	PhD (B.S); MA(S.N)
3. Occupation	What was their occupation at the time of the study?	Junior Lecturer (S.N); Senior Lecturer (B.S)
4. Gender	Was the researcher male or female?	Female (S.N); Male (B.S)
5. Experience and training	What experience or training did the researcher have?	Researcher reflexivity
Relationship with participants		
6. Relationship established	*Was a relationship established prior to study commencement?* .	None
7. Participant knowledge of the interviewer	*What did the participants know about the researcher? (e.g. personal goals, reasons for doing the research).*	Participants received an information sheet describing researcher, study purpose, voluntary participation and confidentiality
8. Interviewer characteristics	*What characteristics were reported about the interviewer/facilitator? (e.g. Bias, assumptions, reasons and interests in the research topic)*	Researcher reflexivity
**Domain 2: Study design**		
Theoretical framework		

Information Classification: General

9. Methodological orientation and Theory	*What methodological orientation was stated to underpin the study? (e.g. grounded theory, discourse analysis, ethnography, phenomenology, content analysis).*	Resilience theory
Participant selection		
10. Sampling	*How were participants selected? (e.g. purposive, convenience, consecutive, snowball)*	Purposive sampling
11. Method of approach	*How were participants approached? (e.g. face-to-face, telephone, mail, email)*	professional associations, social media platforms, and professional networks
12. Sample size	*How many participants were in the study?*	15
13. Non-participation	*How many people refused to participate or dropped out? Reasons?*	0
Setting		
14. Setting of data collection	*Where was the data collected? (e.g. home, clinic, workplace)*	Online via Microsoft Teams
15. Presence of non-participants	*Was anyone else present besides the participants and researchers?*	No
16. Description of sample	*What are the important characteristics of the sample? (e.g. demographic data, date)*	Results
Data collection		
17. Interview guide	*Were questions, prompts, guides provided by the authors? Was it pilot tested?*	Semi-structured guide developed from literature, ProQOL and Resilience Theory; expert reviewed and pilot tested
18. Repeat interviews	*Were repeat interviews carried out? If yes, how many?*	No
19. Audio/visual recording	*Did the research use audio or visual recording to collect the data?*	Audio recorded
20. Field notes	*Were field notes made during and/or after the interview or focus group?*	Reflexive memos maintained during analysis

Information Classification: General

21. Duration	*What was the duration of the inter views or focus group?*	30-45 minutes
22. Data saturation	Was data saturation discussed?	Recruitment continued until no new themes emerged
23. Transcripts returned	*Were transcripts returned to participants for comment and/or correction?*	No
**Domain 3: analysis and findings**		
Data analysis		
24. Number of data coders	How many data coders coded the data?	Initial coding undertaken by the first author; themes refined through discussions with supervisory team
25. Description of the coding tree	*Did authors provide a description of the coding tree?*	Themes and subthemes described in Results
26. Derivation of themes	*Were themes identified in advance or derived from the data?*	Inductive coding informed by study aims and Resilience Theory
27. Software	*What software, if applicable, was used to manage the data?*	NVivo 14
28. Participant checking	*Did participants provide feedback on the findings?*	No
Reporting		
29. Quotations presented	*Were participant quotations presented to illustrate the themes/findings? Was each quotation identified? (e.g. participant number)*	Illustrative quotations with participant IDs and line numbers
30. Data and findings consistent	*Was there consistency between the data presented and the findings?*	Yes
31. Clarity of major themes	*Were major themes clearly presented in the findings?*	Yes
32. Clarity of minor themes	Is there a description of diverse cases or discussion of minor themes?	Yes

Information Classification: General
